# Early Detection of Pulmonary Embolism in a General Patient Population Immediately Upon Hospital Admission Using Machine Learning to Identify New, Unidentified Risk Factors: Model Development Study

**DOI:** 10.2196/48595

**Published:** 2024-07-30

**Authors:** Ori Ben Yehuda, Edward Itelman, Adva Vaisman, Gad Segal, Boaz Lerner

**Affiliations:** 1 Department of Industrial Engineering and Management Ben-Gurion University of the Negev Beer-Sheva Israel; 2 Education Authority Chaim Sheba Medical Center Faculty of Health Science and Medicine, Tel-Aviv University Tel-Aviv Israel; 3 Cardiology Division, Rabin Medical Center Petach-Tikva Israel

**Keywords:** pulmonary embolism, deep vein thrombosis, venous thromboembolism, imbalanced data, clustering, risk factors, Wells score, revised Genova score, hospital admission, machine learning

## Abstract

**Background:**

Under- or late identification of pulmonary embolism (PE)—a thrombosis of 1 or more pulmonary arteries that seriously threatens patients’ lives—is a major challenge confronting modern medicine.

**Objective:**

We aimed to establish accurate and informative machine learning (ML) models to identify patients at high risk for PE as they are admitted to the hospital, before their initial clinical checkup, by using only the information in their medical records.

**Methods:**

We collected demographics, comorbidities, and medications data for 2568 patients with PE and 52,598 control patients. We focused on data available prior to emergency department admission, as these are the most universally accessible data. We trained an ML random forest algorithm to detect PE at the earliest possible time during a patient’s hospitalization—at the time of his or her admission. We developed and applied 2 ML-based methods specifically to address the data imbalance between PE and non-PE patients, which causes misdiagnosis of PE.

**Results:**

The resulting models predicted PE based on age, sex, BMI, past clinical PE events, chronic lung disease, past thrombotic events, and usage of anticoagulants, obtaining an 80% geometric mean value for the PE and non-PE classification accuracies. Although on hospital admission only 4% (1942/46,639) of the patients had a diagnosis of PE, we identified 2 clustering schemes comprising subgroups with more than 61% (705/1120 in clustering scheme 1; 427/701 and 340/549 in clustering scheme 2) positive patients for PE. One subgroup in the first clustering scheme included 36% (705/1942) of all patients with PE who were characterized by a definite past PE diagnosis, a 6-fold higher prevalence of deep vein thrombosis, and a 3-fold higher prevalence of pneumonia, compared with patients of the other subgroups in this scheme. In the second clustering scheme, 2 subgroups (1 of only men and 1 of only women) included patients who all had a past PE diagnosis and a relatively high prevalence of pneumonia, and a third subgroup included only those patients with a past diagnosis of pneumonia.

**Conclusions:**

This study established an ML tool for early diagnosis of PE almost immediately upon hospital admission. Despite the highly imbalanced scenario undermining accurate PE prediction and using information available only from the patient’s medical history, our models were both accurate and informative, enabling the identification of patients already at high risk for PE upon hospital admission, even before the initial clinical checkup was performed. The fact that we did not restrict our patients to those at high risk for PE according to previously published scales (eg, Wells or revised Genova scores) enabled us to accurately assess the application of ML on raw medical data and identify new, previously unidentified risk factors for PE, such as previous pulmonary disease, in general populations.

## Introduction

### Pulmonary Embolism Diagnosis—a Challenge Faced by Modern Medicine

Pulmonary embolism (PE) occurs when a blood clot enters and blocks (either fully or partially) the pulmonary veins, usually because of a dislodged thrombosis in the deeper veins of the lower limbs (also termed “deep vein thrombosis” [DVT]), which is an aspect of the venous thromboembolism (VTE) phenomenon [1]. After myocardial infarction and cerebral stroke, PE is the third most common cause of death from cardiovascular diseases. The worldwide financial burden of PE is immense. Reports have found the estimated cost of a thromboembolic event in the United States to be US $3000 to US $10,000, the total annual cost related to VTE to be US $33,000, and that of VTE complications to be more than US $40,000, with the US health care system’s total annual expenses due to VTE reaching US $12 billion [2,3]. This cost is not significantly different in Europe. By considering the general population admitted to the hospital, we can help reduce mortality rate of later-diagnosed patients, employ early prevention means, and better use hospital resources [4], thereby alleviating this financial burden and improve health care.

### Early Identification of Patients at Risk of PE

Along the VTE continuum, there is a clear correlation between the rapidity of diagnosis and the risk of mortality [5]. Various risk factors and symptoms of PE have been suggested over the years. In addition to previous cases, which increase the risk of recurrence, other patient characteristics associated with this condition include being older than 70 years, active malignancy, congestive heart failure, chronic obstructive pulmonary disease, systolic arterial hypertension, presenting with tachypnea, and right ventricular hypokinesis on echocardiography [1]. New or worsening shortness of breath, chest pain, or sustained hypotension without an obvious alternative cause [5], as well as cigarette smoking, diabetes, obesity, and any type of immobility, is also a contributing factor [6]. For suspected PE, hospital wards commonly use scoring methods designed to predict plausibility, such as the Wells score and the revised Geneva score, to decide whether to send a patient for confirmatory tests [7]. However, it is difficult to compare the Wells score for PE with scoring methods that rely entirely on medical facts, and the revised Geneva score, which is based entirely on clinical variables rather than the experience of the physician, also requires a reasonable pretest probability or initial suspicion of PE. Moreover, while the former score is considered subjective, the latter is ineffective in safely reducing the number of unnecessary computed tomographic scans [8].

In current practice, after a clinical probability assessment, the diagnostic workup should be tailored to the severity of the clinical presentation based on whether the patient’s condition is hemodynamically stable or unstable. In patients with hemodynamic stability, the diagnosis of PE should follow a sequential diagnostic workup potentially consisting of D-dimer blood testing and (if necessary) multidetector computed tomographic angiography (CTA; considered to be the gold standard diagnostic procedure for PE) or ventilation-perfusion scanning (mainly in cases where CTA is contraindicated). Patients with a high pretest probability for PE should not undergo D-dimer blood testing but should immediately be sent for a confirmatory test [5].

However, many cases of PE are diagnosed late or misdiagnosed, and there are 2 main reasons for this. The first is that risk factors, which are presumably known, have yet to be proven connected to the disease, and the second is that the diagnostic methods currently used in hospitals are limited; they are not accurate, safe, or sufficiently available for routine use. Over the years, studies on PE have focused mainly on improving its diagnosis by analyzing CTA images, and when considering early diagnosis, the focus was on risk factors and symptoms that may indicate an elevated or high risk for PE.

### Prediction Tools for Patients at Risk of PE

In the recent years, research on prediction tools for PE has studied several aspects of the disease, for example, by suggesting clinical prediction rules (eg, the Wells and revised Genova scores) [9] and comparing them [10], predicting PE in clinically suspected patients [11,12], predicting adverse outcomes in PE [13], and the application of statistical means for prediction [11-13].

In addition, with the advent of artificial intelligence (AI) as a predictive tool, several studies applied AI to the diagnosis of PE. For example, 1 study [14] used AI and non-AI tools to predict PE-imaging outcomes based on the patient’s electronic medical record to provide a patient-specific risk score for those referred for computed tomography. Other studies used feature engineering and an artificial neural network to predict PE [15] or clustering to differentiate typical clusters of patients [16]. Note, however, that all of these studies, like many others, were performed using the data of patients during their hospital stay.

In a recent study, Ryan et al [17] devised an AI algorithm that predicts the chances for PE during hospitalization. The algorithm used data from laboratory tests conducted during the patient's hospital stay and achieved performance values of 81% sensitivity, 35%-70% specificity, and an area under the curve of 0.67-0.85. However, their approach does not meet a challenge faced by clinicians, which is the need to make a diagnosis immediately upon a patient’s emergency department presentation when clinical data are only partially available and laboratory test results are still unavailable. Moreover, if we use the performance values above to calculate the positive predictive value (PPV), that is, precision, measuring the chances of a true-positive result of all results predicted as positive (which was not reported), we obtain a value of 1.8%, which reflects too many false positives (ie, on average, only 1.8 of 100 subjects predicted as patients with PE are real patients). A system based on such an algorithm will fail to become a practical diagnostic tool, since it did not address the very high inherent imbalance in the problem in favor of non-PE (ie, the very low prevalence of 309 patients with PE among the 60,297 patients tested [17]).

Another recent study by Shen et al [18] concentrated on data collected during the first 3 hours of hospitalization, mainly vital signs and laboratory tests results, from high-risk patients (patients who recently had heart failure, have a history of specific diseases, or currently have cancer, risk factors that are also included in the Wells and Geneva screening scores). Although we acknowledge the potential value of this study, we question its applicability to the general population of hospital admissions. We note that making PE predictions for patients who have already met screening score requirements (and failing to detect those who have not), are at high risk for PE, and collecting data after patients’ hospitalization are not relevant when the aim is to screen the general population immediately upon admission. Second, although predictions are based on patients who have already met screening score requirements and are at high risk for PE and based on data after patients’ hospitalization, the PPV performance reported is only of 20%, again due to a failure to consider the imbalanced inhospital data (3% PE prevalence), which also makes the models proposed by the authors less practical for PE screening on hospital admission.

In contrast, this study focuses on the identification of patients at high risk for PE in the general patient population presenting at emergency departments, using only their demographic and medical history data, prior to the generation of any inhospital data, and using 2 methodologies to tackle the imbalance in PE prevalence in hospital admission data.

### The Aim of This Study

Our study had three main goals: (1) to identify new PE risk factors, among those available to the medical staff upon hospital admission, that may have been overlooked; (2) to accurately predict PE as early as possible upon patient admission, as assessed by all performance measures (including the PPV), in order to create a practical diagnostic tool for PE; and (3) to group patients using the newly identified and already known risk factors in order to find subgroups of patients who are at higher risk of PE upon hospital admission. That is, the main goals of this study were not only to establish an AI tool for early diagnosis of PE but also to identify new, previously unidentified risk factors for PE that clinicians should have in mind when caring for their general population of patients—and not only those already defined as having high risk of PE—and to use these risk factors to identify informative subgroups of patients at risk of PE upon hospital admission. The fact that we did not restrict our patients to those with high risk for PE according to previously published scales (eg, Wells and revised Geneva scores) enabled us to accurately assess the application of our AI algorithms on raw medical data.

There are 3 novelties in our study. First, unlike previous studies, our study concentrates on the data of a general population of patients admitted to tertiary care and not just those with a high probability or suspicion of PE. Since straightforward modeling of PE is prone to imbalance due to the low disease prevalence among the admitted patient population, where only a minority of patients admitted to the hospital will later be diagnosed with PE, our second novelty is in proposing 2 methodologies aimed at overcoming the highly imbalanced scenario that provide reasonable PPV values. The third novelty is in a new performance measure we apply to a conventional clustering algorithm to identify clusters that maximize the minority-to-majority (PE to non-PE) ratio and thereby help us focus on PE risk factors and groups of patients at risk on hospital admission from the additional angle of a clustering measure.

In the sections that follow, we describe the data used in our study and how we addressed the imbalance challenge (“Methods” section), our modeling methods (“A Methodology for Meeting Our 3 Main Goals on Hospital Admission” section), and derived results (“Results” section) before summarizing and proposing future research directions (“Discussion” section).

## Methods

In this section, we present the patient cohort and database, along with our data understanding and preparation methods. Our methods for tackling imbalance, which are described in Multimedia Appendix 1 [19-34], use the features and past diagnoses that we identify in this section as being most effective in differentiating PE from control patients.

### Patients

The data were obtained by Sheba Medical Center (SMC) using the MDClone ADAMS Platform, maximizing collaboration with synthetic data while maintaining patient privacy and maximizing data use [35]. Balancing patient privacy, legal, compliance, and security issues often hinders individuals, teams, and organizations from working together to share data. However, the MDClone ADAMS Platform overcomes these common obstacles by allowing users to access and share data and information across both internal and external entities with synthetic data safely and securely. It provides data not only for research purposes but also for real-time clinical applications, which ensures the high reliability of the extracted data. Several publications have validated the usage of MDClone-generated synthetic data for epidemiological studies. In 1 study, Foraker et al [36] thoroughly compared the synthetic and real data of septic patients and did not find differences in the statistical conclusions derived. Benaim et al [37] also validated the application of MDClone-generated synthetic data in 5 observational studies and concluded that predictability was highly preserved. In this study, 2600 patients diagnosed with PE and 53,250 patients with non-PE diagnoses (controls) were represented by their demographic, clinical, laboratory test results, and medical history data.

### Ethical Considerations

The synthetic data were sampled from the real-world, raw data distribution such that the statistical characteristics of their distribution replicate those of the true distribution and do not contain identifiable information included in the real data. Clinical events in the synthetic samples are slightly and randomly shifted in time to avoid the possibility of de-anonymizing the data. To protect patient privacy, the SMC database consists of synthetic data generated from the data of all patients who were hospitalized in the center’s internal medicine departments between 2008 and 2020. Patients’ data were retrieved, and the MDClone system was used after the Sheba Medical Center institutional review board approval of this study (7864-20-SMC).

### Data Understanding and Preparation

We suggest a model capable of predicting PE in a patient upon his or her admission to the hospital, based only on the data available in the patient’s electronic medical record before admission. We focus exclusively on data available prior to emergency department admission, as these are the most universally accessible data worldwide. This approach allowed us to overcome the challenge stemming from variability in hospital-specific clinical decisions and routines, which could limit the broader applicability of our findings. We collected demographics, comorbidities, and chronic medications data for 2568 patients with PE and 52,598 control patients. While patient records for the age and sex variables were complete, the BMI variable was frequently missing; thus, we removed records with missing BMI values. This left us with the data of 1942 patients with PE (PE=1) and 44,697 control patients (PE=0). Table 1 provides some descriptive statistics for age, sex, and BMI for the patients with PE and controls. Since the 2 populations were of unequal sample sizes and variances, to check significance, we applied a Welch’s *t* test (unequal variance 2-tailed *t* test). According to this test, patients with PE were significantly older (mean 69.4, SD 16.8 vs mean 65.3, SD 17.5; *P*<.001) and significantly more obese (mean 27.2, SD 5.9 vs mean 26.8, SD 5.3; *P*<.001). Moreover, female patients were diagnosed with PE significantly more often than male patients (1097/1942, 56.5% vs 845/1942, 43.5%; *P*<.001). Note, however, that our data were obtained for all patients admitted to the internal department of SMC, and, thus, case subjects and control subjects were not matched, which may explain some of the differences between the 2 groups.

Since the time of past diagnoses is not always recorded in hospital records, and sometimes it is even based on patient memory, we ignored this parameter and treated each of these clinical events as a binary variable indicating whether a patient had received this diagnosis in the past. Then we merged 19 past diagnoses into 9 categories of diagnostic “families,” as is frequently done. In addition to the clinical motivation, this merging also helped us unify sparse diagnoses into denser categories. We treated past PE as a stand-alone category and did not associate it with the thrombosis category, as would be customary, because it is a known risk factor for PE, and we wanted to examine it as a “stand-alone” diagnosis in our model. Also, we converted the only chronic medication variable existing in our data, the registration date of a patient receiving treatment with anticoagulants, into a binary variable indicating whether the patient had received such treatment in the past. Table 2 shows the prevalence of the 9 categories of past diagnoses and that of anticoagulant use for the 2 patient groups. Except for the gastrointestinal past diagnosis, the differences in prevalence between the 2 groups for all past diagnoses are statistically significant according to the Welch’s *t* test.

**Figure 1:**
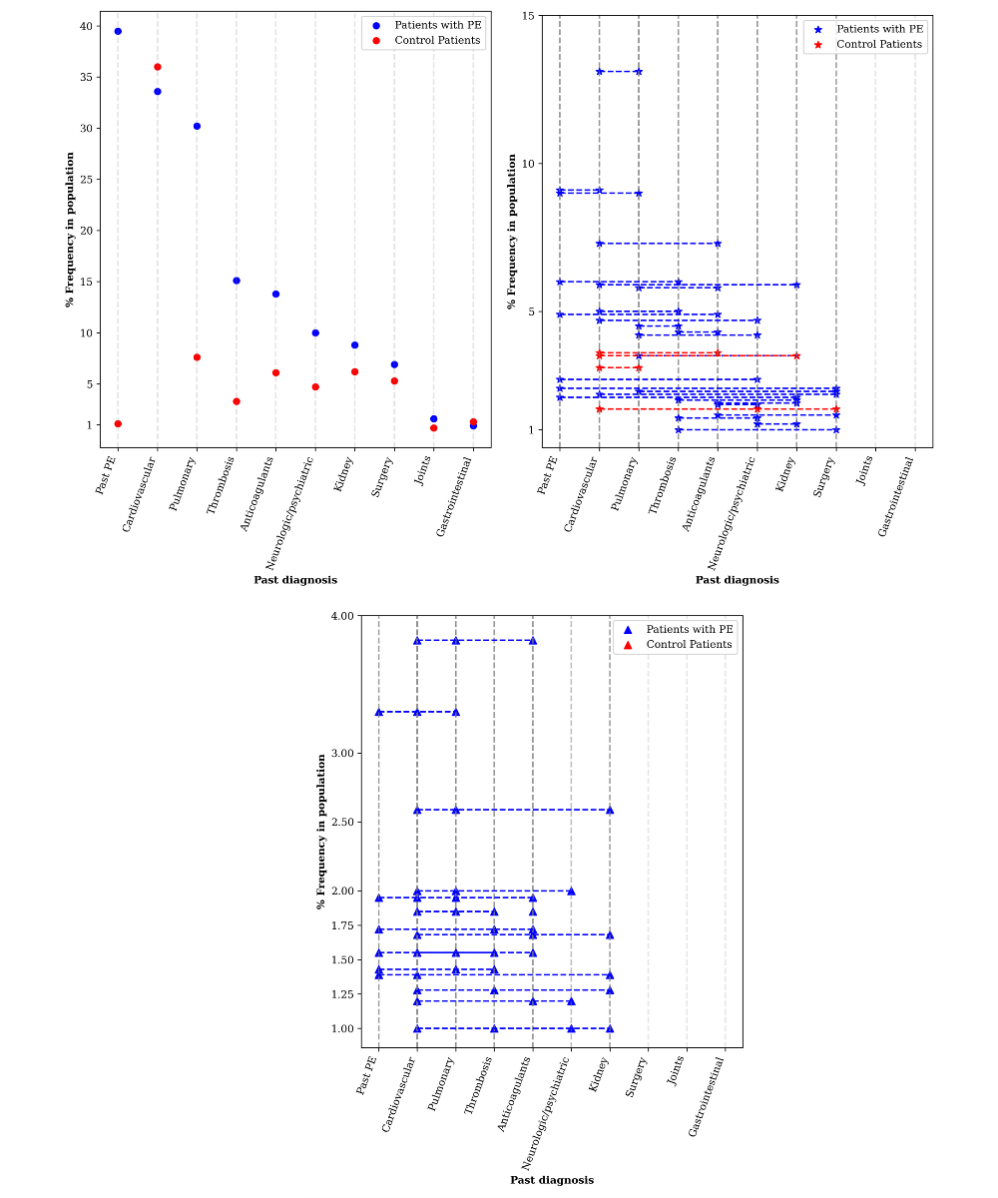
Prevalence for a single past diagnosis or medication in each of the study populations (top left), a pair of past diagnoses or medications in each population (top right), and a triple past diagnosis or medication in each population (bottom). PE: pulmonary embolism.

Using the PrefixSpan algorithm, which is designed to discover sequential patterns in sequence databases [38], we looked for common combinations of 2 or more past diagnoses (plus the single variable indicating the use of anticoagulants) in each of the PE and control patients. In general, we found that the patients with PE had a richer background of past diagnoses than the control patients, with only 15.7% (305/1942) of them not having any past diagnosis, compared with 46.8% (20,918/44,697) of the control patients (*P*<.001). In addition, we found that the 2 patient groups showed a high incidence of cardiovascular disease in the past. As for the combinations, pulmonary and cardiovascular background diagnoses were the most common, with 13.1% (254/1942) of the patients with PE having both (compared with only 3.1% [1386/44,697] of the control patients), while the combination of cardiovascular and past PE affected 9.1% (177/1942) (447/44,697; 1% control), pulmonary and past PE affected 9% (175/1942; 447/44,697, 1% of the control group), and cardiovascular and anticoagulant usage affected 7.3% (142/1942; 1609/44,697, 3.6% of the control group). We also identified 16 combinations of 3 variables and 1 combination of 4 variables (anticoagulants, pulmonary, cardiovascular, and past PE) affecting more than 1% (20/1942) of patients with PE. It is notable that the control patients did not have a single combination of 3 or more variables in more than 1% of the population. Figure 1 presents the prevalence (%) of past diagnoses and chronic medications for PE and control patients for single diagnoses, pairs of past diagnoses, and combinations of 3 past diagnoses. The past diagnoses identified here served as the basis for patient representation when tackling imbalance in training and testing PE classifiers (Multimedia Appendix 1).

**Table 1:** Descriptive statistics for patients with pulmonary embolism (PE) and control patients.

Variable		Sex distribution			*P* value
		PE^a^	Control		
**Sex (%)**					<.001
	Male	43.5	55.4		
	Female	56.5	44.6		
Age (years), mean (SD)		69.4 (16.8)	65.3 (17.5)		<.001
BMI, mean (SD)		27.2 (5.9)	26.8 (5.3)		.003

^a^PE: pulmonary embolism.

**Table 2:** Composition and prevalence of past diagnosis categories and anticoagulant use in our database.

Category	Past diagnosis	Prevalence (%)		*P* value
		PE^a^	Control	
Surgery	Past surgery documentation Past surgery procedure	6.9	5.3	<.01
Pulmonary	Pneumonia Pleural effusion Chronic obstructive pulmonary disease Restrictive lung disease	30.2	7.6	<.001
Thrombosis	Deep vein thrombosis Coagulopathies	15.1	3.3	<.001
Cardiovascular	Ischemic heart disease Atrial fibrillation or atrial flutter Congestive heart failure	33.6	36	.03
Kidney	Chronic kidney disease	8.8	6.2	<.001
Gastrointestinal	Inflammatory bowel disease	0.9	1.3	.07
Joints	Rheumatoid arthritis	1.6	0.7	<.001
Neurologic or psychiatric	Dementia Mental disorders Mood disorders Psychosis	10.0	4.7	<.001
Past PE	Past PE	39.5	1.1	<.001
Anticoagulants	Anticoagulants	13.8	6.1	<.001

^a^PE: pulmonary embolism.

### A Methodology for Meeting Our 3 Main Goals on Hospital Admission

The 3 main goals of this study were to identify new PE risk factors, among those available to the medical staff upon hospital admission that may have been overlooked, to accurately predict PE as early as possible upon patient admission, and to group subgroups of patients who have the newly identified together with known risk factors and are at higher risk of PE. To achieve the first goal, we identified important features (previous diagnoses in the patient’s medical records) according to the classifier, validated this identification using an ablation study, and focused the analysis on important individual diagnoses that may be considered potential risk factors. To achieve the second goal, we used performance measures dedicated to imbalanced scenarios to estimate the ability of the 2 methodologies we suggested (Multimedia Appendix 1) and establish a common benchmark that would enable the comparison of the 2 methods. To achieve the third goal, we clustered the population on hospital admission to identify some homogenous clusters that maximize the minority-to-majority (PE to non-PE) ratio and then focused on risk factors that established these risk groups.

### Feature Importance—Initial Identification of Potential Risk Factors

We used a random forest (RF) consisting of a collection of tree-structured classifiers, each trained on bootstrapped samples of the training data, that randomly searches across subsets of the 13 input variables (the 10 in Table 2 plus the 3 in Table 1) to determine the forest’s trees. The output of the classifier is a majority vote of the trees. To maximize its performance, we adjusted 3 hyperparameters of the RF: maximum depth—the longest path possible from a tree root to a tree leaf; minimum sample split—the minimal number of samples required to split an internal node; and the split criterion—either the Gini impurity or the information gain [39,40]. Our prior research demonstrates the accuracy and efficiency of the RF classifier in several clinical domains [41-45]. To increase confidence in the classifiers obtained by methods 1 and 2 (a description of the methods is provided in Multimedia Appendix 1), we repeated the evaluation of the methods over 10 data permutations and reported the averaged performance.

The RF model measures the contribution (ie, importance) of its input variables (features) to classification using the Gini index, measuring the chance of misclassifying a random record based on the a priori probabilities of each class in a particular split in a tree [39]. However, evaluation over 10 data permutations yielded 10 lists of feature importance for each method. Therefore, to determine feature importance, we proposed to evaluate the statistical difference between 10 feature lists of both methods using a Friedman-Nemenyi test [46], as described in Multimedia Appendix 2.

### Accurate PE Prediction Under the Imbalanced Scenario

**Figure 2:**
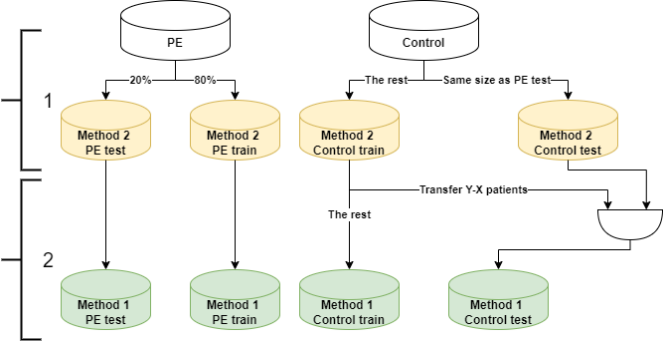
Division into training and test sets to compare methods 1 and 2. X and Y are the numbers of patients equal to 20% of the PE and control sets, respectively. PE: pulmonary embolism.

To perform an in-depth comparison of the performance of methods 1 and 2, we needed to evaluate them using the same data both in the training and tests sets, because method 1 uses more control subjects than method 2. Therefore, we evaluated the methods using a positive (PE) class measure, the true-positive rate (TPR), and divided the data into training and test sets in 2 steps (Figure 2):

1. We created the data sets for method 2 by creating a balanced test set: 20% of the PE subjects and the same number of control subjects and left the remaining subjects for training.
2. We created the data sets for method 1 by keeping the same PE test and training sets used in method 2. Then, we balanced the remaining control patients (beyond those used in method 2) between the control training and test sets to include 80% and 20% of the original control data set, respectively.

To focus the evaluation of methods 1 and 2 on their performance on patients with PE, we compared the TPR values of the 2 methods using a univariate 2-tailed *t* test [47].

### Clustering to Informative Homogeneous Subgroups for PE

To add to our study additional angle on the characteristics of patients at risk for PE on hospital admission, we wanted to find as homogeneous subgroups as possible for PE using a different performance measure than those already used. To achieve this goal, first we relaxed the categorization of the 19 indications into the 9 categories we created earlier whether there were specific past diagnoses that had not been detected in the process performed so far that may be considered as potential risk factors for PE. Second, we established different possible feature representations for patients based on all combinations of features (past diagnoses and demographics data). Third, we suggested to maximize a performance measure that accounts for the percentages of patients with PE in a clustering scheme’s clusters to simultaneously solve 2 patient representation problems that are usually solved separately, and their optimization is based on results of each other. These are the optimal feature representation and clustering scheme (number of clusters). Using this measure and the *K*-means algorithm, we clustered patients while considering different patient representations (combinations of possible feature representations and possible clustering schemes with *K*={2, 5}) to derive that which yields clusters that maximize the percentage of patients with PE compared with the PE prevalence in the data (1942/46,639, 4.16%).

## Results

We evaluated our methodology under the imbalanced scenario according to five criteria: (1) Gini-based feature significance, (2) classification performance measures, (3) ablation-based feature significance, (4) TPR-driven maximization, and (5) identification of potential risk factors.

### Gini-Based Feature Significance

Using 10 data permutations, we applied methods 1 and 2 (Figures S1 and S2 in Multimedia Appendix 1) to predict PE and obtained 10 feature importance lists for the 13 features for each method. Testing each method separately using a Friedman-Nemenyi test, past PE was ranked the most significant by both methods, and age, BMI, pulmonary, and past thrombosis (Tables 1 and 2) were ranked not significantly differently than past PE by both methods, as well as anticoagulant usage for method 1 and sex for method 2. The other 6 features (ie, past diagnoses: surgery, cardiovascular, kidney, gastrointestinal, joints, and neurologic or psychiatric) were ranked significantly differently than past PE.

### Classification Performance Measures

Tables 3 and 4 present confusion matrices for methods 1 and 2, respectively, and Table 5 presents performance measures of the best model based on the 7 most important features for each method (derived as described in the “Gini-Based Feature Significance” section) averaged over 10 data permutations. The tables demonstrate that method 1 achieved higher performance values on most measures than method 2 (and similar true-negative values) in most cases except for precision and the *F*_1_-score. The very low precision value and very high negative predictive values of method 1 suggest that the decision threshold that was selected by this method is low, allowing more subjects to pass the threshold, among them (because of the imbalance) more controls that are thus incorrectly predicted as PE, reducing precision. However, on the other hand, this “low” threshold guarantees that almost all subjects that are identified as controls are indeed controls, increasing the negative predictive values to almost a perfect value. This “low” threshold also ensures predicting more patients with PE as PE and fewer as controls, both of which increase the TPR. These mechanisms represent a trade-off in the calculation of the main performance measure, as is expected in imbalanced scenarios.

**Table 3:** Method 1—confusion matrix.

	True negative	True positive	Total predicted
Predicted negative	0.83	0.01	0.84
Predicted positive	0.13	0.03	0.16
Total true	0.96	0.04	1

**Table 4:** Method 2—confusion matrix.

	True negative	True positive	Total predicted
Predicted negative	0.44	0.16	0.6
Predicted positive	0.06	0.34	0.4
Total true	0.5	0.5	1

**Table 5:** Performance measures (the values of measures for the best method are italicized).

Measure	Accuracy	Precision	TPR^a^	TNR^b^	NPV^c^	*F*_1_-score	GM^d^
Method 1	*0.86*	0.19	*0.75*	0.86	*0.99*	0.30	*0.80*
Method 2	0.78	*0.85*	0.68	*0.88*	0.73	*0.76*	0.77

^a^TPR: true-positive rate.

^b^TNR: true-negative rate.

^c^NPV: negative predictive values.

^d^GM: geometric mean.

### Ablation-Based Feature Significance

While the selection of features was Gini-based, we wanted to back this selection based on classification performance and an ablation study [48]. For each method, we compared a classifier based on the 7 features that are the union of the important features identified for both methods (past PE, age, BMI, pulmonary, thrombosis, anticoagulants, and sex), a classifier that is based on each method’s 6 selected features, and 6 classifiers in which each is missing 1 of the 6 important features of a method. We trained each classifier on 10 data permutations and examined statistical difference between the geometric mean values of the classifiers using the validation set and the Friedman-Nemenyi test for each method separately. We observed that only those classifiers missing past PE, pulmonary, or thrombosis were significantly worse in their geometric mean value than the full classifier (the one that included all 7 important features). We also observed that there was no significant difference between classifiers of the 2 methods. This analysis reinforces the results presented in the “Gini-Based Feature Significance” section about the significance of past PE, pulmonary, and thrombosis to PE identification.

### TPR-Driven Maximization

To examine whether one method was superior to the other on the positive (PE) group when using the same patients with PE in training and testing, we compared the TPR values of the best models produced by the 2 methods using 10 data permutations and the univariate 2-tailed *t* test. We found that there was no significant difference between the 2 methods regarding the TPR results at 95% CI.

### Identification of Potential Risk Factors

To deepen our understanding of PE on hospital admission, we relaxed the categorization of the 19 indications into the 9 categories we created earlier (in the “Data Understanding and Preparation” section) to examine whether there were specific past diagnoses that had not been detected in the process performed so far, which may be considered as potential risk factors for PE. Simultaneously, we also attempted to find as homogeneous a group as possible of patients with PE. We hoped that such a group would be characterized differently than other groups, hinting at potential PE risk factors.

**Figure 3:**
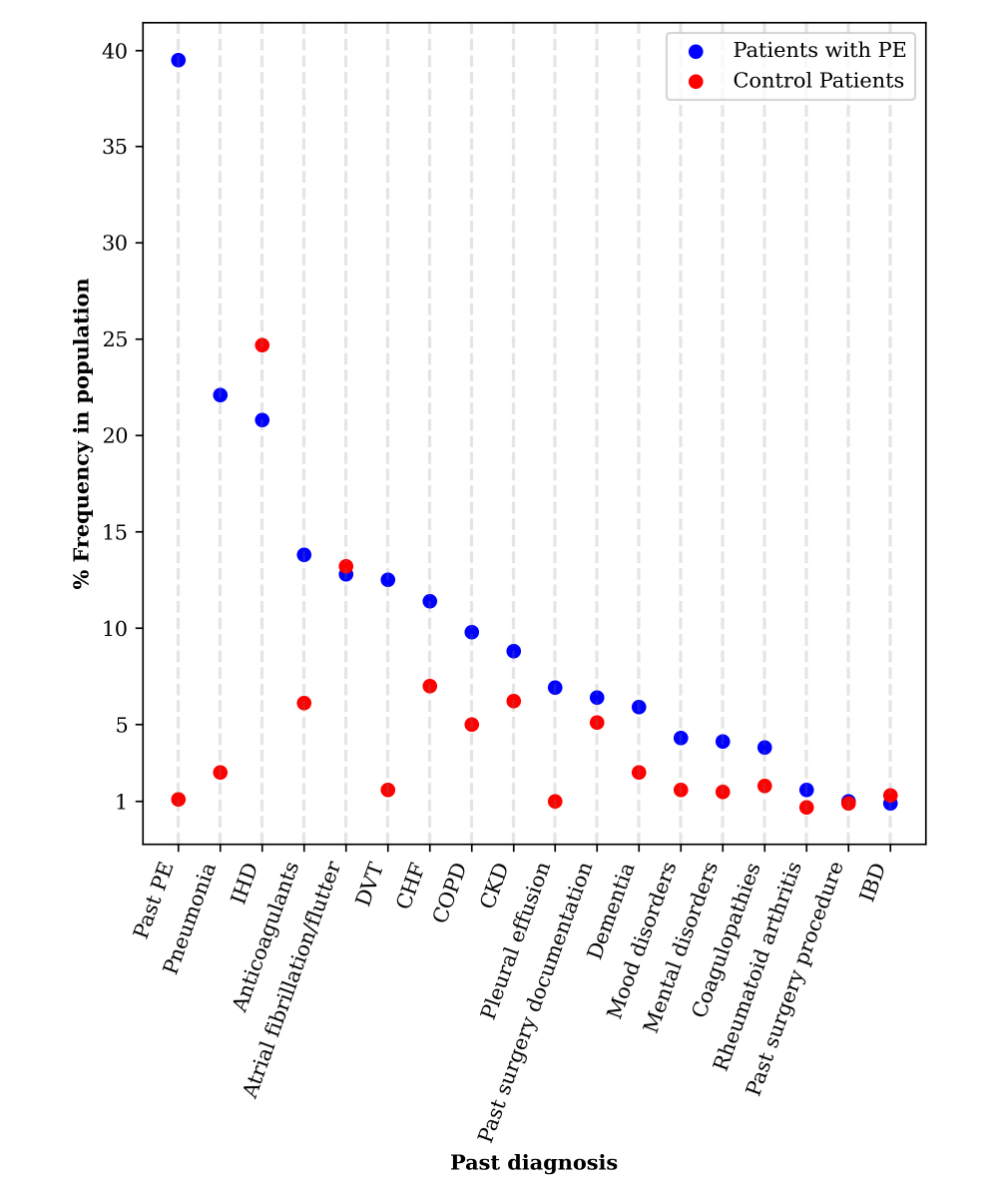
Prevalence of an original past diagnosis or medication in each of the study populations. CHF: congestive heart failure; CKD: chronic kidney disease; COPD: chronic obstructive pulmonary disease; DVT: deep vein thrombosis; IBD: inflammatory bowel disease; IHD: ischemic heart disease; PE: pulmonary embolism.

First, we examined the prevalence of each of the past diagnoses existing in our data for each of the patients with PE and control patients. We found that the prevalence of several of these past diagnoses, such as past PE, pneumonia, and DVT, was higher in patients with PE than in control patients (Figure 3). On the other hand, the only past diagnosis having an incidence higher than 1% in control patients compared with patients with PE was ischemic heart disease.

Next, to ensure our ability to find the most homogeneous group of patients with PE, we clustered, using the *K*-means algorithm, patient representations using different combinations of the features [49]. The only features we chose to omit from the clustering process were those with a frequency below a 2% threshold in both populations: past surgery procedure, rheumatoid arthritis, inflammatory bowel disease, psychosis, and restrictive lung disease. Therefore, we were left with 18 possible features for patient representation: 3 demographic (age, sex, and BMI), 14 past diagnoses, and 1 chronic medication (anticoagulants). Because the prevalence of patients with PE in our data was very low (1942/46,639, 4.16%), we looked for a cluster or more where the percentage of patients with PE was significantly higher than this threshold.

**Figure 4:**
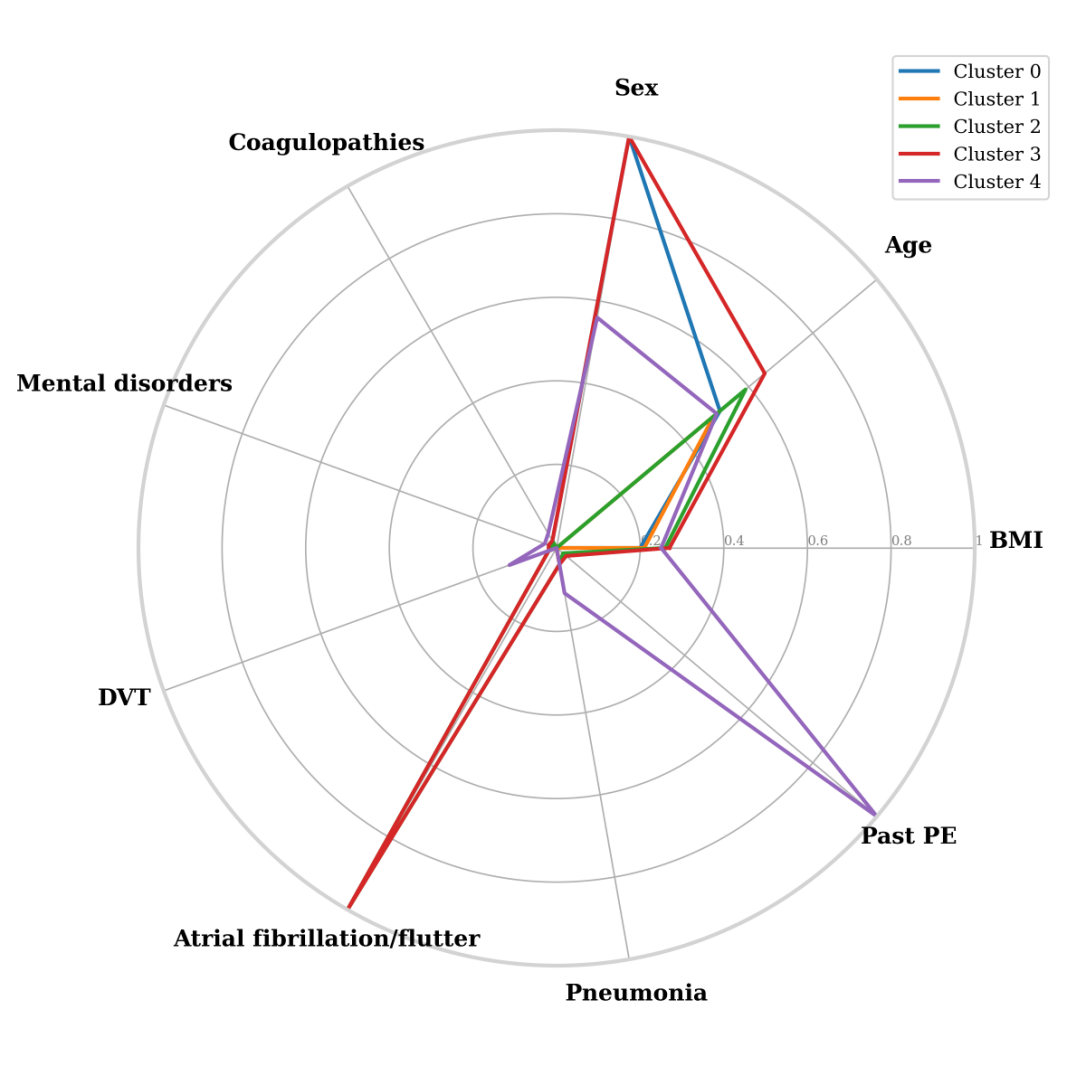
Radar plot of characteristics of the first clustering scheme. A point of a particular cluster on the axis of any feature constitutes the average normalized value (between 0 and 1) for the patients in the cluster. DVT: deep vein thrombosis; PE: pulmonary embolism.

After simultaneously considering possible clustering schemes (*K*={2, 5}) and patient representations based on a combination of features, we found 2 cluster schemes that yielded interesting results. The first scheme (Figure 4), which yielded the cluster with the highest frequency of patients with PE (705/1120, 63%; cluster 4 in Table 6), is a 5-cluster scheme in which each patient is represented by sex, age, BMI, and past PE, past pneumonia, history of atrial fibrillation or atrial flutter, past DVT, history of mental disorders, and history of coagulopathies. We see that cluster 4, which is the most PE-populated cluster of this scheme, represents a profile that is based on both demographic and medical history features, suggesting complex relations between such characteristics that are nontrivial and need further exploration. Cluster 4 contained 1120 patients (1120/46,639, 2.4% of all patients): 705 were patients with PE (705/1942, 36.3% of all patients with PE in our data) and only 415 were controls. Prominent characteristics of this cluster compared with the others are that all its patients had a past diagnosis of PE (see Table 7), a relatively high incidence of past DVT (12% compared with 2% in any of the other clusters) and past pneumonia (11% compared with 4% or less in any of the other clusters), and a mediocre incidence of mental disorders, and they more often took anticoagulants. Patients in this cluster were equally mixed by sex. The other clusters had only a small incidence of such past diagnoses and were composed of single-sex patients (Table 7). That is, this clustering scheme made a clear distinction between a single small, but very PE dominant, cluster 4, and 4 other non-PE clusters that are mainly dominated by demographics (sex and age) and BMI characteristics. Besides showing past PE and DVT as risk factors, which are known to clinicians (see, eg, the study by Scarvelis and Wells [50]), patients in cluster 4 also reveal past pneumonia history as a factor, which is evidence of a new risk factor of PE.

**Figure 5:**
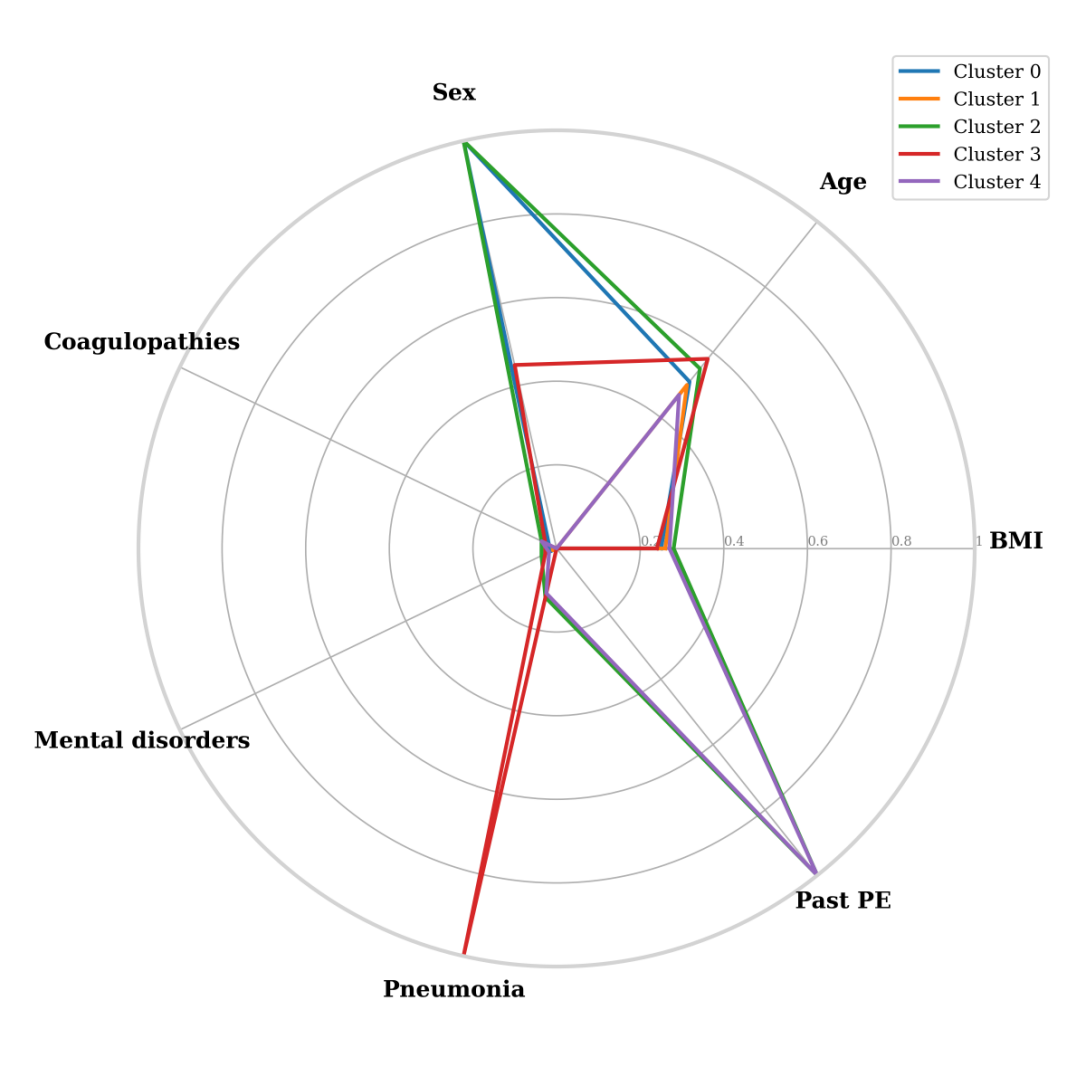
Radar plot of characteristics of the second clustering scheme. A point of a particular cluster on the axis of any feature constitutes the average normalized value (between 0 and 1) for the patients in the cluster. PE: pulmonary embolism.

The second clustering scheme (Figure 5), which resulted in 2 clusters (clusters 2 and 4) with a prevalence of patients with PE of 61% (427/701 and 340/549) or more (Table 8), was a 5-cluster schema in which patients were represented by sex, age, BMI, and past PE, past pneumonia, history of mental disorders, and use of anticoagulants. All patients in these 2 clusters had a past diagnosis of PE and a higher incidence of past diagnosis of pneumonia and usage of anticoagulants (Table 9), higher BMI values, and, interestingly, they were divided into only women (cluster 2) or only men (cluster 4). Also, cluster 3, which has a relatively high frequency of patients with PE—20% (301/1470) compared with 4.16% in the full database (Table 8), is characterized by all patients having a past diagnosis of pneumonia. That is, the chances of developing PE according to this scheme are mainly related to either past PE and past pneumonia or, to a lesser degree, only past pneumonia. Although the incidence values of a past diagnosis of mental disorders and usage of anticoagulants were low, these 2 variables were chosen for patient representation by both clustering schemes, because their contribution to patient segregation was significant in maximizing the cluster PE frequency we optimized in the clustering. The atrial fibrillation or flutter diagnosis was selected only by the first clustering scheme, producing clusters with either all subjects or none of them with this diagnosis, as this partition helped maximize the target measure. Finally, in addition to past PE, which is arguably the most important variable for marking patients with suspected PE, past diagnoses of pneumonia and DVT may also lead to PE. Thus, we recommend overattention to admitted patients with these past diagnoses.

**Table 6:** PE^a^-control prevalence in the clusters of the first clustering scheme (the highest PE prevalence for a cluster is italicized).

Cluster, n	Relative size	Patients, n		PE/total
		PE	Control	
0	0.38	564	17,046	0.03
1	0.47	426	21,395	0.02
2	0.07	106	3179	0.03
3	0.06	141	2662	0.05
4	0.02	705	415	*0.63*

^a^PE: pulmonary embolism.

**Table 7:** Average feature values in the clusters of the first clustering scheme.

Cluster, n	Sex	Age (years)	BMI	Past PE^a^	Pneumonia	Atrial fibrillation/flutter	DVT^b^	Mental disorders	Coagulopathies
0	1	65.5	26.5	0	0.03	0	0.02	0.02	0.02
1	0	63.2	26.8	0	0.03	0	0.02	0.01	0.01
2	0	72.0	27.7	0.02	0.04	1	0.02	0.01	0.02
3	1	75.6	27.9	0.03	0.04	1	0.02	0.02	0.02
4	0.56	64.3	27.6	1	0.11	0	0.12	0.03	0.04

^a^PE: pulmonary embolism.

^b^DVT: deep vein thrombosis.

**Table 8:** Descriptive statistics of the clusters of the second clustering scheme^a^.

Cluster, n	Relative size	Patients, n		PE/total
		PE^b^	Control	
0	0.42	510	19,161	0.03
1	0.52	364	23,884	0.02
2	0.02	427	274	*0.61*
3	0.03	301	1169	0.20
4	0.01	340	209	*0.62*

^a^Italicized values indicate the high prevalence of patients with pulmonary embolism in clusters 2 and 4 compared with the prevalence of 4.16% of these patients in the cohort.

^b^PE: pulmonary embolism.

**Table 9:** Average value of each feature in each cluster of the second clustering scheme^a^.

Cluster, n	Sex	Age (years)	BMI	Past PE^b^	Pneumonia	Mental disorders	Coagulopathies
0	1	66.7	26.7	0	0	0.02	0.02
1	0	64.2	27.0	0	0	0.01	0.01
2	*1*	67.3	27.8	*1*	0.12	0.04	0.04
3	0.45	69.3	26.5	0	*1*	0.03	0.03
4	*0*	63.0	27.5	*1*	0.11	0.02	0.04

^a^All patients of clusters 2 and 4 (see Table 8), clusters that are mostly populated by patients with PE, have a past diagnosis of pulmonary embolism and higher incidence of past diagnosis of pneumonia and usage of anticoagulants. While cluster 2 composes of only women relatively old, cluster 4 includes only men relatively young (italicized values).

^b^PE: pulmonary embolism.

## Discussion

We are not the first to address the potential benefits in applying AI to PE, as AI does not require additional clinician input or cause workflow disruption by automatically screening a broad inpatient population [14-18]. These studies reported the application of AI in interpreting chest images [14] or their accompanying radiology reports [51] and in using laboratory test results and vital signs [17,18] of already diagnosed patients with PE. In addition, these studies often targeted PE at patients who are already at elevated risk of PE [18].

To better understand and predict the occurrence and diagnosis of PE, we have suggested tools that may be clinically available on patient hospital admission—the earliest point in time to model PE in the hospital. Also, we addressed the entire population of patients presenting to a tertiary hospital, without narrowing them to only those with high probability of PE, as required by the Wells or revised Genova scores, to screen the full admitted population and not only those with higher risk of PE. Using 13 years of data from around 50,000 patients with PE and those with no PE, we considered the medical history of patients presenting in a hospital, where their prior chance of being diagnosed with PE was 4%. To create reliable models, we suggested 2 methods to address this highly imbalanced clinical scenario. The first empirically sets a classifier decision threshold to account for the minority-to-majority ratio in the imbalanced data, and the second uses an ensemble of balanced classifiers evaluating the PE class versus equal-sized disjoint portions of the imbalanced non-PE class. To identify significant predictive past diagnoses, we considered diagnoses in 2 resolutions, separately and categorized, and proposed a nonparametric statistical test to consolidate feature importance lists obtained over data permutations. An ablation study validated results based on the test. Lists produced by the 2 methods were almost identical, and classifiers trained using them provided similar performance, validating the correctness of both methods. Past diagnoses of PE, pulmonary diseases, and thrombosis, together with age, sex, BMI, and usage of anticoagulants contributed to 80% accuracy in early identification of both patients with PE and those with no PE, with no statistical difference between the methods.

Although only 4% of the patients in our data set had a definitive diagnosis of PE, we identified, by joint optimization of feature representation and a clustering scheme, subgroups comprising more than 60% PE-positive patients. These subgroups facilitated a more in-depth analysis to identify demographic and past diagnosis characteristics, demographic-clinical relations, and potential PE risk factors. Some of our findings are not conventionally considered. For example, in medical practice, a past diagnosis of pulmonary disease on admission is related to a chronic respiratory disease displaying respiratory symptoms, but we show here that this may also be an indication of PE. This is an important clinical pearl that should prompt clinicians worldwide, with and without access to AI predictive algorithms.

In summary, despite the high imbalance in the data and the very early stage of modeling, our prediction and clustering models were both accurate and informative in identifying patients at high risk for PE, at the time of hospital admission, before a patient is even seen by a hospital doctor. The main advantage in our findings, which is relevant to every health care professional, is the fact that applying AI enables whole-patient-population analysis without the need to adhere to previously published criteria (eg, Wells or revised Genova scores), which are not sensitive enough [52], or to limit its use to high-risk patients. In the case of PE, this approach defines previous pulmonary disease, whether with past PE or alone, as a new, significant risk factor to consider.

A limitation of our research is working with data, which, due to privacy restrictions, were synthesized from real data. The difference between real data and synthetic data derived from real data is that the synthetic data include times of diagnosis and measurements that are randomly shifted to up to a year before or after the real time and thus could not be verified. Therefore, in our study, the existence of past diagnoses was expressed using binary variables (whether a diagnosis exists or not) and not by the times of diagnosis, which is vital information that could have contributed greatly to the models.

Our future research will use real data and also assimilate data from the emergency department (validating and amending, as necessary, the admission model) and then the internal ward, whenever data are collected, which may necessitate the use of dynamic modeling. In addition, while our second method was applied to a balanced test set, it should be further examined on a nonbalanced database that reflects the true PE prevalence. Finally, beyond being the basis for predictive and informative models of PE, we see our workflow as suitable for other diseases, especially since in almost all of them, an imbalanced scenario is involved.

## Appendix

Multimedia Appendix 1 Methods developed for imbalanced data.

Multimedia Appendix 2 A statistical test to consolidate different recommendations on feature importance.

## Abbreviations

AI: artificial intelligence
CTA: computed tomographic angiography
DVT: deep vein thrombosis
ML: machine learning
PE: pulmonary embolism
PPV: positive predictive value
RF: random forest
SMC: Sheba Medical Center
TPR: true-positive rate
VTE: venous thromboembolism
