# Supplementary material for: Early Detection of Pulmonary Embolism in a General Patient Population Immediately Upon Hospital Admission Using Machine Learning to Identify New, Unidentified Risk Factors: Model Development Study
Source: J Med Internet Res. 2024 Jul 30;26:e48595. doi: 10.2196/48595 (PMC11322683; doi:10.2196/48595)

The challenge with imbalanced data is that the minority class cannot be represented and, thus, be learned well, which undermines classifier performance. To tackle the class imbalances scenario, we combine a method that overcomes imbalance with a performance measure that is sensitive to the imbalance. Several known methods for handling class imbalance balance the populations of the classes, usually by either up-sampling the minority class or down-sampling the majority class, apply penalty/loss terms on majority classes, and learn models that maximize performance measures that are indifferent to the imbalance [37],[38],[39],[40],[41],[42],[43]. While accuracy is a good performance measure when handling balanced data, it leads to misleading results in cases of imbalance and therefore should not be considered in these cases [44]. For example, assume the data include 95.8% of the patients negative for PE and 4.2% of the patients positive for it (as in this study), and also assume a classifier that aims at maximizing accuracy and therefore chooses (by setting a very high threshold for the class posterior probability) to classify all instances as negative for PE. Then we will achieve very high accuracy of approximately 95.8% but fail to classify all positive patients. Another performance measure, the area under the receiver operating characteristic (ROC) curve (AUC) accounts for both the true positive rate (TPR) and false positive rate (FPR). The TPR is the percentage of positive instances correctly classified as belonging to the positive class, while the true negative rate (TNR) is the percentage of negative cases correctly classified as belonging to the negative class. The AUC summarizes the performance for all possible cutoff thresholds applied to the probability to belong to the positive class [45] (each such threshold may represent a different majority-to-minority ratio); however, in an imbalance problem like ours, the optimal model would fit the threshold that matches the imbalance ratio in the data, otherwise it will not be accurate for the minority class [46]. Therefore, like the accuracy measure, the AUC should not be considered in training and validating a classifier in an imbalanced scenario.

Here, we deal with the imbalance by both proposing and evaluating two new methods and using appropriate performance measures for imbalance problems. The first method adjusts the decision threshold on the classifier output (posterior probability of PE). When dealing with balanced data, for a two-class classification task, a threshold of 0.5 reflects equal prior probabilities. However, for imbalanced data like ours, a threshold of 0.5 is not suitable. To demonstrate this, we first trained an RF model with a decision threshold of 0.5. The model identified the control patients in 99.2% of the cases but only identified the PE patients in 34% of the cases. Therefore, we determined the threshold to correspond to the minority-to-majority class ratio in order to give the minority class instances a higher chance to be classified as PE and allow the classifier to achieve higher accuracy for both classes. Then, we evaluated performance using the geometric mean (GM) which better suits class imbalance classification problems, because it is indifferent to the class sizes [47]. The GM is the square root of the product of TPR and TNR [48]. Since the GM considers the accuracies of the two classes equally, selecting a classifier that maximizes the GM is ideal for class imbalance classification problems [47]. Two other performance measures suitable for imbalanced problems are the F1 measure, although it prefers the positive class over the negative class, and the Matthew's correlation coefficient (MCC) which depends on the class size ratio [49].

Method 1, in which an RF classifier is trained based on the empirical evaluation of decision thresholds using the GM measure, is presented in Figure S1. A description of the steps in Method 1 follows:

After splitting the PE and control data into training (80%) and test (20%) sets, we split each (PE and control) training set into five random datasets [5-fold cross-validation (CV 5): four sets for inner training and one set for validation].

After combining PE and control datasets for each of five stratified inner training and validation sets, we trained an RF for each parameter configuration of max depth of a tree classifier (between 10 and 30), minimum number of samples needed for a split (2, 3, or 4), and split criterion (Gini impurity or information gain).

Each RF trained using some parameter configuration was validated by the corresponding validation set and the GM for each decision threshold (between 0.02 and 0.06; a range evaluated empirically around the minority-to-majority class ratio).

The RF model (parameters and threshold) achieving the highest average GM (over the five validation sets) was selected.

Based on the optimal model (parameters and decision threshold), we trained an RF model using the entire training set and reported our results on the test set.

**Figure S1.** Resolving imbalance by determining the PE-control decision threshold (method 1).


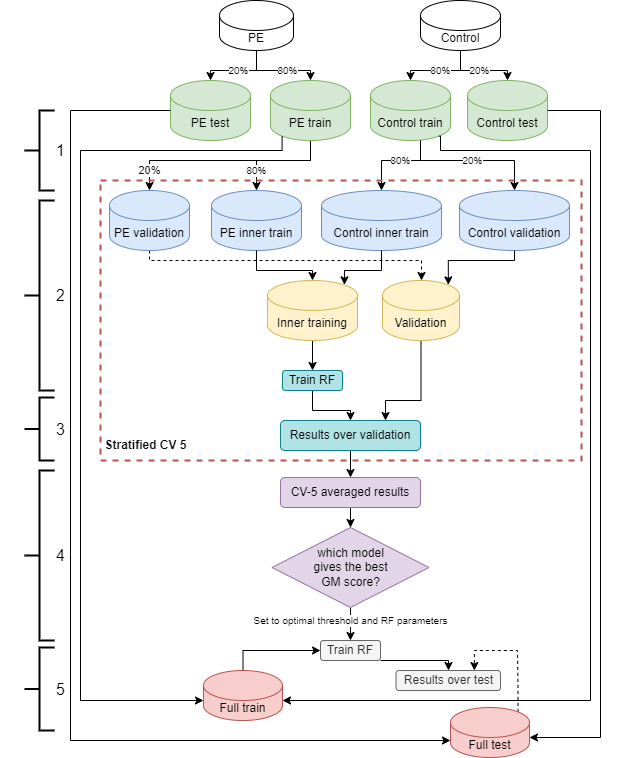


In Method 2, the majority class is down-sampled to reach a balanced scenario that can be evaluated by a decision threshold of 0.5 [42],[50]. To balance the data, we can up-sample by using synthetic or duplicate records of patients who are positive for PE or down-sample the control group (negative for PE) by choosing a random group of instances equal in size to the PE-positive group [41],[42],[43],[47],[50],[51]. Because we had many PE-negative patients and did not want to create synthetic data that cannot be fully relied upon, we chose to down-sample the control group. In addition to the benefit of reaching a balanced scenario, down-sampling of the majority class left us with many unused instances from the control group, which allowed us to train classifiers based on the minority class, with another control group each time; after averaging, this ensemble provides more reliable results. The training of each such classifier using a balanced set provided higher accuracy for the minority class, with just minimal deterioration of accuracy for the majority class, than if the classifiers had learned from the imbalanced dataset. Although we also report results for the GM, the measure chosen to examine the quality of the model was the F1 measure, which combines precision and recall (TPR). Precision (also designated as sensitivity) is the ratio of the number of positive instances correctly classified as positive to the total number of instances classified as positive. The F1 score was chosen, because in a balanced scenario like that of the second method, we wanted to maximize predictivity of the positive group more than the negative group, and both recall and precision promote this [52].

Method 2, in which an ensemble of RF classifiers is trained based on balanced sets, is presented in Figure S2. A description of the steps in Method 2 follows:

After splitting the PE data into test (20%) and training (80%) sets, and the control data into test (equal in size to the PE test set) and training sets (all remaining control patients), from the control training set we down-sampled *S* non-overlapped random datasets, *S* equals to the ratio between the control and PE training sets.

We split each of the *S* control training datasets and the PE training set into five random datasets (CV 5; see Method 1).

After combining PE and control datasets for each of five stratified inner training and validation sets for each of the *S* control training datasets, we trained an RF for each parameter configuration of max depth of a tree classifier (between 10 and 30), minimum number of samples needed for a split (2, 3, or 4), and split criterion (Gini impurity or information gain).

For each of the *S* control training datasets, we validated each RF classifier that was trained using some parameter configuration by the matched validation set and F1 measure.

For each of the *S* control training datasets, we selected the RF parameter configuration achieving the highest average F1 measure (over the five validation sets).

We trained *S* RF classifiers using the entire training set and each of the selected *S* parameter configurations.

We reported test results as averaged over this *S* classifier ensemble and the test set.

**Figure S2.** Resolving imbalance by training a classifier ensemble using PE-control balanced data sets (method 2).


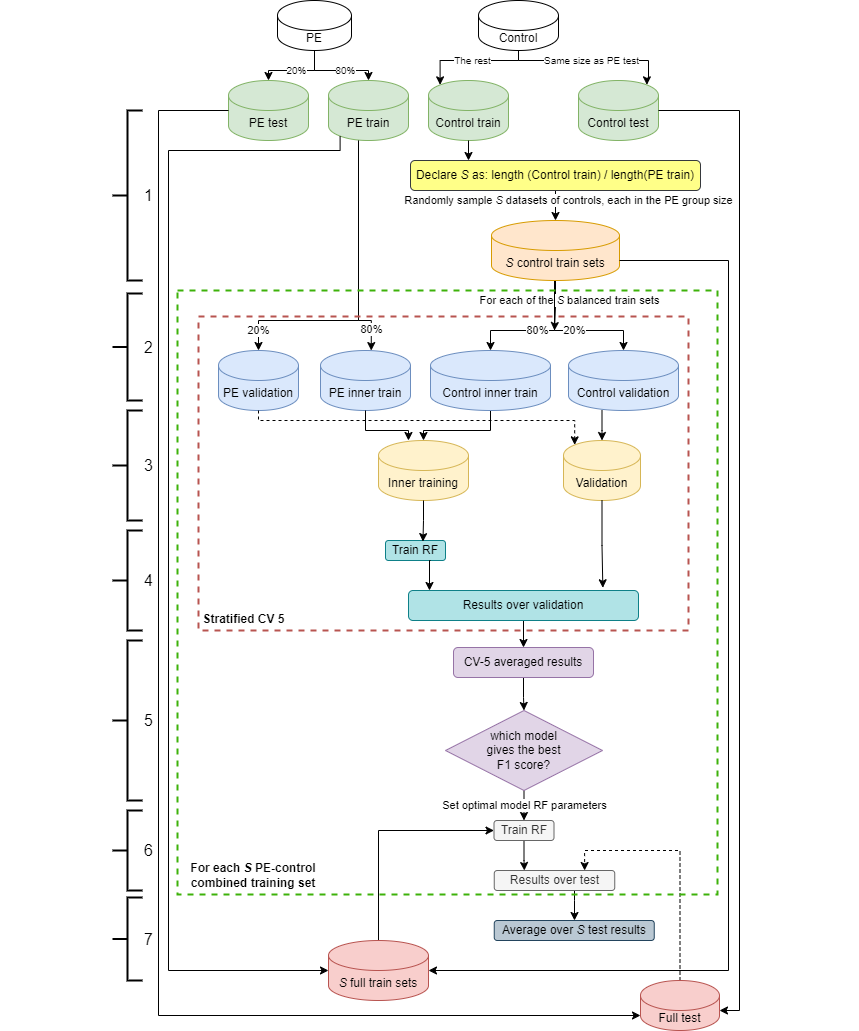

Supplement: Multimedia Appendix 1 [file jmir_v26i1e48595_app1.docx]
