# Supplementary material for: Early Detection of Pulmonary Embolism in a General Patient Population Immediately Upon Hospital Admission Using Machine Learning to Identify New, Unidentified Risk Factors: Model Development Study
Source: J Med Internet Res. 2024 Jul 30;26:e48595. doi: 10.2196/48595 (PMC11322683; doi:10.2196/48595)
Supplement: Multimedia Appendix 2 [file jmir_v26i1e48595_app2.docx]

Originally, this test was designed to measure significant differences between classifier accuracies validated on several databases. The Friedman test is a non-parametric equivalent of the repeated-measures ANOVA. It ranks the classifiers for each database separately: the best performing classifier receives a rank of one, and the worst receives a rank of $k$ (i.e., the number of classifiers). The test compares the average (over databases) ranks of classifiers under the null hypothesis, which states that all classifiers are equivalent and, therefore, their ranks should be equal. If the F-distributed Friedman statistic has a higher value than a critical value, we reject the null hypothesis that the classifiers are the same and apply the Nemenyi test comparing the classifiers to each other. The performance of two classifiers is significantly different if the corresponding average ranks differ by at least the critical difference,

$$CD = q_{\alpha}* \sqrt{\frac{k\left( k +1 \right)}{6N}} ,$$

where $N$ is the number of databases, and critical value $q_{\alpha}$ is based on the Studentized range statistic divided by $\sqrt{2}$. Here, we propose replacing 1) classifier accuracy with feature importance and 2) databases with lists of feature importance to apply a powerful statistical test to consolidate different recommendations on feature importance in a single decision.
